# Knowledge, attitude and practice (KAP) of health providers towards safe abortion provision in Addis Ababa health centers

**DOI:** 10.1186/s12905-019-0835-x

**Published:** 2019-11-14

**Authors:** Endalkachew Mekonnen Assefa

**Affiliations:** School of Medicine, Department of Obstetrics and gynecology, Addis Ababa University College of Health Sciences (AAU-CHS), P. O. Box 9086, Addis Ababa, Ethiopia

**Keywords:** Mid-level providers, Safe abortion care

## Abstract

**Background:**

Unsafe abortion remains a reality for many Ethiopian women and will remain so until safe abortion is more accessible across the country. The house of representatives of Federal Democratic Republic of Ethiopia (FDRE) revised the abortion law and Ministry of Health (MoH) of FDRE developed a revised technical and procedural guideline for safe abortion services in Ethiopia; emphasizing the need to increase knowledge and practice of health service providers on safe abortion care (SAC) and access to safe terminations of pregnancy at high standard and quality.

**Methods:**

A facility based descriptive cross-sectional study using structured self-administered questionnaire was conducted between July and August 2015. A total of 405 mid-level providers (MLPs) including midwives, clinical nurses and health officers were included from 30 randomly selected health centers in Addis Ababa. SPSS version-21 was used for data entry, cleaning and analysis. The results were presented using frequency tables, percentages, means, Odds ratio and 95% confidence limits.

**Results:**

Among 405 MLPs 71.9% knew the definition of abortion in the in Ethiopia context, 81.5% participants were familiar with the revised abortion law. 53.1% of respondents had adequate knowledge on safe abortion care and working for 3–5 years (AOR 3.1 with CI 1.6, 5.7) and midwives (AOR = 2.9 with CI 1.8, 4.7) had better knowledge on abortion. Only eighty-three (20.5%) of MLPs were trained on safe abortion and among them sixty-eight (81.9%) were practising/used to practice safe abortion services. Half of respondents gave post abortion family planning methods. 54.1% respondents had positive attitude towards safe abortion. MLPs’ who had adequate knowledge on safe abortion care (AOR 2.02, 95% CI 1.3–3.1) and male providers (AOR 1.6, 95% CI 1.04–2.4) were more likely to have positive attitude towards safe abortion. MLPs who had adequate knowledge on abortion 3.4 times (CI of 95% =1.1–10.6) were more likely to practise safe abortion care.

**Conclusion:**

The majority claimed to know the current abortion law; however, many failed to understand the specific provisions of the law. Type of profession and years of experiences were important in explaining providers’ knowledge related to abortion. Being male and having the knowledge significantly influenced providers’ attitude toward safe abortion. Knowledge related to abortion also influenced the practice of SAC. Efforts to improve mid-level as well as other health care providers’ knowledge on abortion are necessary, for example, through pre−/on-service training.

## Introduction

Nearly half of all abortions (21.6 million) worldwide are unsafe, and nearly all unsafe abortions (98%) occur in developing countries [1–4].

Unsafe abortion remains a reality for many Ethiopian women and will remain so until safe abortion is more accessible across the country. An estimated number of 382,500 induced abortions were performed in Ethiopia; among induced abortion, only 27% of abortions (some 103,000 abortions) had safe procedures performed in health facilities [5, 6].

The health risks of abortion depend on whether the procedure is performed safely or unsafely [7, 8]. According to WHO, unsafe abortion remains one of the four leading causes of pregnancy-related death, disabilities and injuries around the world, along with hemorrhage, infection and high blood pressure in connection with childbirth [1, 9]. Every day, more than 128 women die from complications of unsafe abortion [1, 7, 9].WHO estimates that in Eastern Africa, unsafe abortion accounts for one in seven maternal deaths [1, 10]. Ethiopia has the fifth highest number of maternal deaths in the world: One in 27 women die from complications of pregnancy or childbirth annually [1, 11].

Where some services are available, limited resources, lack of adequate trained health provider, lack of equipment, inadequate provision of contraceptives, lack of awareness, cultural stigma, and over all poor socio-economic status further limit women’s access to quality care. In such environment providers may have little training and experience with methods of termination of pregnancy, which can translate into poor quality information, and counseling [1, 8, 12].

Little is known about knowledge, attitude and practice of mid-level health providers towards safe abortion provision in Ethiopia. Therefore, this study attempts to assess health providers’ knowledge and attitude especially after the change of Penal Code, and to assess their practise of safe abortion services. Since there is no similar published study conducted in our country; it can contribute a lot as baseline information for future studies, planners and policy makers on mid-level providers (clinical nurses, health officers and midwives) related to safe abortion services. Also it will give a great benefit to reproductive communities in general.

## Methods

### Study design and setting

A facility based descriptive cross-sectional study was conducted between July and August 2015 by using a structured self-administered questionnaire. The study was conducted in Addis Ababa, is the capital city of the Federal Democratic Republic of Ethiopia (FDRE). The city is divided into 10 sub-cities and 99 Weredas [13]. In Addis Ababa there are 7 hospitals under the regional Addis Ababa health Bureau and 4 hospitals under Federal government. There are also 88 health centers (including 1 Ebola center) and 760 private clinics of thus, 40 are specialty clinics and hospitals [14]. In the City there are 5415 health care providers in the government facilities; of thus 866 B.Sc. nurses, 1896 diploma nurses, 608 health officers, 349 midwives and 215 all other nurses [15].

Ten strata were formed according to numbers of sub-cities; from each stratum a sample of study health centers were selected by simple random sampling proportional to their numbers of health centers. Sample size of the participants calculated using 30/7 cluster sampling method (which is WHO recommendation and can be used for non-vaccine related researches) [16]. To avoid design effect, since multi-stage cluster sampling used, the sample size multiplied by 2 and the final sample size was 420.From each selected health centers fourteen MLPs randomly included.

Mid-level Providers (MLPs) used in this study including nurses (B.Sc. diploma nurses), Health Officers, Midwives (diploma, degree graduates).

Abortion used in this study based on the following definition - termination of pregnancy before fetal viability which is less 28 weeks of gestation according to revised technical and procedural guideline on safe abortion service, and penal code [14, 17].

Mid-levels providers’ knowledge and attitude operationally defined. Knowledge and attitude questions scored and normality plots test (Kolmogorov-Smirnov & Q-Q plot) done on SPSS-21 version. It was found normally distributed. Providers ‘who scored above the mean on knowledge questions considered had adequate knowledge on safe abortion. Providers’ who scored above the mean on attitude questions considered had positive attitude towards safe abortion (see attached Additional file 1).

### Data collection and analysis

The self-administered questionnaire, in English version and it was translated back to Amharic and again to English to confirm the correctness of the translation. Fourteen MLPs who were working as full time employee, were chosen randomly from each health centers by considering the homogeneity of health care providers. The data collection was conducted by the principal investigator and four data collectors and completeness was checked daily. The questionnaire was pre-tested prior to data collection in another health facilities to ensure the data quality. SPSS version- 21 used for data entry, cleaning and analysis. The results are presented using frequency tables, percentages, means, odds ratio and 95% confidence limit. In addition to significant variables, selected variables (age, sex, marital status and religion) were included in the logistic model which affected KAP of mid-level providers in other studies.

### Variables

There were four outcome variables. The first was Knowledge about current abortion law and revised technical and procedural guideline on safe abortion services in Ethiopia. The second and third outcomes were attitude toward safe abortion and practice of safe abortion services respectively. The last variable was factors which affect knowledge, attitude and practice of MLPs’ on safe abortion.

### Ethical consideration

After getting ethical clearance from Addis Ababa University College of health sciences department of obstetrics and gynecology research publication committee (MF/Gyn/127/2007) and Addis Ababa Health Bureau IRB (AAHB/6995/227), support letters written to each sub-city and sampled health centers. Written consent took from each participant. Participant’s involvement in the study was on voluntary basis and participants who wished to quit their participation at any stage of study informed to do so without any restriction. Confidentiality maintained at all levels of the study.

## Results

Of the 420 self-administrative questionnaires distributed, 405 were completed and returned, giving a response rate of 96.4%. Among the respondents 245 (60.5%) were females, 132 (32.6%) were younger than 25 years of old. The mean age was 27.04 years (SD ± 5.16), range (15–55 years). About 50.6% were nurses and 32.8% had work experience of 1–3 years (Table 1).

**Table 1:** Frequency distribution of selected socio -demographic characteristics of health care providers, Addis Ababa, August 2015

Characteristics *N* = 405		Frequency	Percent (%)
Sex	Male	160	39.5
	Female	245	60.5
Age	< 25 years	132	32.6
	25–29 years	185	45.7
	30–34 years	48	11.9
	≥ 35 years	40	9.9
Religion	Orthodox	282	69.9
	Muslim	52	12.8
	Protestant	54	13.3
	Catholic	17	4.2
Marital status	Never married	251	62
	Married	134	33.1
	Others ^a^	20	4.9
Profession	Diploma nurses	149	36.8
	B.Sc. nurses	56	13.8
	Midwife diploma	100	24.7
	Midwife degree	31	7.7
	Health officer	69	17
Years of professional experiences	< 1 year	74	18.3
	1–3 years	133	32.8
	3-5 Years	102	25.2
	5-10 years	65	16
	> 10 years	31	7.7

^a^Others include: widowed, separated and cohabited

### Knowledge of respondents related to abortion

Regarding to definition of abortion, 291 (71.9%) of the respondents knew the definition as it defined in the revised abortion law and federal ministry of health of Ethiopia (FMoH) guideline termination of pregnancy before fetal viability (< 28 weeks) and 89.1% said they knew what safe abortion means (Table 2).

**Table 2:** Mid-level health care providers’ knowledge on definition of abortion, safe abortion and procedures, revised abortion law and post abortion care, Addis Ababa, August 2015

Characteristics	No. (%)
Definition of abortion *N* = 405	
Termination of pregnancy < 20 weeks from last normal menstrual cycle (LNMP)	79 (19.5)
Termination of pregnancy < 24 weeks LNMP	13 (3.2)
Termination of pregnancy < 28 weeks LNMP	291 (71.9)
I don’t know	22 (5.4)
Knew safe abortion *N* = 405	
Yes	361 (89.1)
No	44 (10.9)
Types of procedures they knew^a^ *N* = 361	
Manual vacuum aspiration (MVA)	274 (75.9)
Using mifepristone and misoprostol	288 (79.8)
Dilation and curettage (D & C)	209 (57.9)
Evacuation and curettage (E & C)	179 (49.6)
Familiar with the revised abortion law *N* = 405	
Yes	330 (81.5)
No	75 (18.5)
Place for terminating pregnancy *N* = 330	
Equipped health facilities that aren’t authorized to perform the procedure with no trained staff	9 (2.7)
Non-Equipped health facilities that aren’t authorized to perform the procedure with no trained staff	9 (2.7)
Equipped health facilities and trained staff authorized to perform the procedure	281 (85.2)
I don’t know	9 (2.7)
Requirement from a woman for termination of pregnancy due to rape or incest *N* = 330	
Requires to submit evidences	104 (31.5)
Aren’t required to submit evidences	190 (57.6)
I don’t know	31 (9.4)
Continuation of pregnancy endangers the life of woman or child in state safe abortion permitted *N* = 330	
Necessarily be in a state of ill health	59 (17.9)
Shouldn’t necessarily be in ill health	229 (69.4)
I don’t know	42 (12.7)
The provider has to secure informed consent for procedure using standard consent form *N* = 330	
True	278 (84.2)
False	52 (15.8)
Know post abortion care *N* = 405	
Yes	347 (85.7)
No	58 (14.3)

^a^Total do not add to 100 because of multiple response

Regarding knowledge on the pregnancy termination procedures, 75.9% were familiar with manual vacuum aspiration (MVA), using mifepristone and misoprostol (79.8%), 57.9% dilation and curettage (D&C), and 49.6 evacuation and curettage (E&C) (Table 2).

Three hundred and thirty (81.5%) of mid-level health care providers were familiar with the revised abortion law, of them, 281 (85.2%) said termination of pregnancy should be performed in equipped health facilities and trained staffs who are authorized to perform procedure (Table 2).

Concerning safe termination of pregnancy according revised law 190 (57.6%) of health care providers’ didn’t require evidences to give termination of pregnancy due to rape or incest. While, 104 (31.5%) said women should submit evidence to get the service even the law said no requirement of evidences. If continuation of pregnancy endangers the women’s health 229 (69.4%) respondents mentioned that women should not be in ill health to get the services even if the pregnancy endangers the women’s health while 59 (17.9%) said they will give the service if she is necessary ill even the law doesn’t require to be necessary ill to get the services (Table 2).

Related to consent for the procedure 278 (84.2%) said the provider should secure informed consent for the procedure using standard consent form. About 85.7% of mid-level health care providers reported that they knew components post abortion care (PAC) (Table 2).

### Attitudes of mid-level health care providers related to abortion

**Fig. 1:**
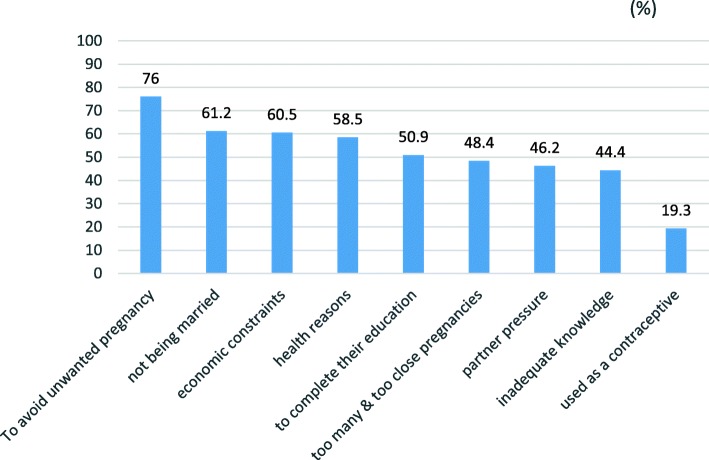
Health care provider attitudes on why women seek abortion, Addis Ababa August 2015. N.B. Total do not add to 100% because of multiple response

Respondents suggested several reasons why women seek abortion. These include to avoid unwanted pregnancy (76.0%), not being married (61.2%), economical constraint (60.5%), health reasons (58.5%), to complete their education (50.9%), too many & too close pregnancies (48.4%), partner pressure (46.2%), inadequate knowledge (44.4%) and 19.3% of the providers believed that women use abortion as contraceptives (Fig. 1).

Two hundred ninety (71.6%) respondents said that they were not comfortable working in a site where termination of pregnancy is performed. Their reasons were against their religion (77.9%), followed by against personal values, not trained on abortion procedure, and outside of the scope of their practice (Table 3).

**Table 3:** Attitudes of health care providers whether they are comfortable working in a place where abortion is done, and on legalization of abortion, Addis Ababa, August 2015

Characteristics	Frequency (%)
Comfortable working in a site where termination of pregnancy is being performed *N* = 405	
Yes	115 (28.4)
No	290 (71.6)
Reasons why not comfortable^a^ *N* = 290	
Outside of the scope of my practice	21 (7.2)
Against my religious practice	226 (77.9)
Against my personal values	126 (43.4)
I didn’t have the opportunity to be trained in abortion technique	49 (16.9)
I don’t know	11 (3.8)
Others	2 (0.6)
Legal abortion should be permitted under any circumstances *N* = 405	
Agree	112 (27.7)
Disagree	244 (60.2)
Neutral	49 (12.1)
Reasons for disagreement^a^ *N* = 244	
My religion doesn’t allow	172 (70.5)
Culturally not accepted	59 (24.2)
It is homicide on the fetus	128 (52.5)
Encourage to have unwanted pregnancies	135 (55.3)
Encourages pre−/extra-marital sex	145 (59.4)

^a^not 100% due to multiple answers

From 405 respondents 244 (60.2%) said abortion should not be legalized under any circumstances. On the other hand, 27.7% said abortion should be legalized under any circumstances (Table 3).

Of the respondents who said abortion should not be legalized as 70.5% said their religion doesn’t allow, 59.4% said it encourages pre−/extra-marital sex. The remaining respondents said it will encourage having unwanted pregnancies, homicide on the fetus and culturally it is not accepted which was 55.3, 52.5% and 24.25 respectively (Table 3).

### Practice of safe abortion care (SAC) among mid-level health providers

Among participants eighty-three said they trained on SAC; of whom, 81.9% said they practiced/practising SAC services (Table 4).

**Table 4:** Mid-level providers training and practice of safe abortion care, and giving post abortion family planning A.A. August 2015

Characteristics	Frequency (%)
Safe abortion training *N* = 405	
Yes	83 (20.5)
No	322 (79.5)
Practice SAC *N* = 83	
Yes	68 (81.9)
No	15 (18.1)
Methods of SAC practiced to termination of pregnancy^a^ *N* = 68	
Medication abortion	65 (95.6)
Manual Vacuum Aspiration (MVA)	50 (73.5)
Dilation & curettage (D&C)	8 (11.8)
Evacuation & curettage (E &C)	6 (8.9)
Offer post abortion family planning N = 405	
Yes	201 (49.6)
No	204 (50.4)
Methods of post abortion family planning^a^ *N* = 405	
Injectables	149 (74.1)
Implants	118 (58.7)
OCP	116 (57.7)
Condom	116 (57.7)
IUD	82 (40.8)
Natural methods	26 (2.9)

^a^not 100% due to multiple answers *SAC* safe abortion care

*OCP* oral contraceptives *IUD* intrauterine device

Concerning methods of pregnancy termination, 95.6%practiced safe abortion service using medication abortion and 73.5% MVA. The others said using D&C, E&C which was 11.8 and 8.9% respectively (Table 4).

Of the total 405 respondents 201 (49.6%) gave post abortion family planning, and frequently they gave injectable (74.1%), implants (58.7%), condom and oral contraceptive pills (OCP) (57.7% for each), Intrauterine device (IUD) (40.8%) and 2.9% natural methods (Table 4).

### Factors associated with knowledge

In general 215(53.1%) of respondents had adequate knowledge (i.e. respondents who scored above the mean score) related to abortion. Out of them 123 (50.2%) of females and 92 (57.5%) of males had adequate knowledge related to abortion (Table 5).

**Table 5:** Factors which had impact on knowledge of MLPs’ on safe abortion, Addis Ababa, August 2015

Variables	Adequate knowledge	Inadequate knowledge	Crude OR (95% CI)	Adjusted OR (95% CI)
	Number (%)	Number (%)		
Profession				
Nurses	87 (42.4)	118 (57.6)	1	
Midwives	81 (61.8)	50 (38.2)	2.197 (1.403–3.440)*	2.875 (1.764–4.687)**
			2.898 (1.627–5.160)*	2.653 (1.451–4.852)**
Health officers	47 (68.1)	22 (31.9)		
Age (Years)				
< 25	51 (38.6)	81 (61.4)	1	
25–29	112 (60.5)	73 (39.5)	2.437 (1.542–3.852)*	2.875 (1.764–4.687)**
				1.299 (0.587–2.876)
30–34	27 (56.3)	21 (43.8)	2.647 (1.276–5.491)*	1.718 (0.694–4.254)
≥ 35	25 (62.5)	15 (37.5)		
Years of experiences				
< 1	27 (36.5)	47 (63.5)	1	
1–3	64 (48.1)	69 (51.9)	1.615 (0.901–2.892)	1.699 (0.899–3.215)
3–5	65 (63.7)	37 (36.3)	3.58 (1.642–5.696)*	2.807 (1.331–5.917)**
≥ 5	59 (61.5)	37 (38.5)	2.776 (1.483–5.195)*	2.724 (1.195–6.211)**

*P < 0.05

**Statistically significant after adjusted for age, sex, marital status, religion, types of profession & years of experiences

Respondents by their age, less than 25 years (38.6%), 25–29 years (60.5%), 30–34 years (56.3%) and 35 years and above had adequate knowledge. From them 35 years and above had better knowledge than others by 2.6 (CI of 95% 1.3–5.5). Also age groups 25–29 years and 30–34 years were more knowledgeable than less 25 years by 2.4 (CI = 1.5–3.9) and 2 times (COR = 1.04–3.99) respectively. However, the same variables were insignificant with adjusted ratio (Table 5).

Among respondents of mid-level health care providers’ midwives (61.8%) and health officer (68.1%) had better knowledge above the mean by 2.2 times (CI = 1.4–3.4) and 2.9times (CI =1.6–5.2) respectively than nurses. The same variable appeared statistically significant after adjustment which was 2.9 (1.764–4.687) and 2.65 (1.5–4.9) respectively (Table 5).

The other variable associated was years of professional experiences, and from providers who worked 3–5 years had better knowledge 3.1 times (CI = 1.6–5.7) than less than 1 year of experience. Also knowledge related to abortion increased 2.8 times (CI = 1.5–5.2) in providers who worked 5 and more years. It was statistically significant after adjustment 2.8 (1.3–5.9) and 2.7 (1.2–6.2) respectively. All other demographic and practice variables didn’t show any significant in explaining changes of knowledge score (Table 5).

### Factors associated to attitude of MLPs towards safe abortion

From respondents 54.1% had positive attitude towards safe abortion. Male had positive attitude towards safe abortion 1.7 times (CI of 95% 1.127–2.536) than females. It was statistically significant after adjustment (adjusted OR = 1.6; CI = 1.04–2.4). The other associated factor was knowledge on abortion which showed MLPs who had adequate knowledge on abortion were favorable towards safe abortion 2.2 (CI = 1.5–3.3.7). It was significant after adjusted 2.02 (1.3–3.1) (Table 6).

**Table 6:** Factors which affect of MLPs’ attitude for safe abortion, Addis Ababa, August 2015

Variables	Positive attitude	Not positive attitude	Crude OR (95% CI)	Adjusted OR (95% CI)
	Number (%)	Number (%)		
Sex				
Female	120 (49.0)	125 (51.0)	1	
Male	99 (61.9)	61 (38.1)	1.69191.127–2.536)*	1.591 (1.044–2.426)**
Knowledge related to abortion				
Adequate knowledgeable	136 (63.3)	79 (36.7)	1	
Inadequate knowledgeable	83 (43.7)	107 (56.3)	2.219 (1.489–3.307)*	2.019 (1.335–3.055)**

*P < 0.05 ** Statistically significant after adjusted for sex, knowledge, age, marital status, education level, religion, years of experiences, knowledge related on abortion

### Factors impact on practice of safe abortion care

Among 405 respondents 68 (16.8%) were currently practicing or used to practise. The only variable showed association was knowledge on abortion; providers who had adequate knowledge related to abortion were better to practise SAC 3.4 times (CI of 95% =1.1–10.6).

## Discussion

405 mid-level providers (MLPs) who were working in thirty health centers of Addis Abeba were their knowledge, attitude and practice and determining factors on safe abortion provision analyzed. Majority knew the definition of abortion in Ethiopian context and safe abortion, familiar with revised abortion law. Nearly three-fourth of participants were not comfortable working in a site where termination of pregnancy performed and only one-fourth of participants agreed on permission of legal abortion under any circumstances. Age, types of profession and years of experience had positive effect on knowledge of safe abortion whereas gender and knowledge related to abortion determine attitude on safe abortion.

To reduce unsafe abortion and its harmful complication Article 551 of the penal code of Federal Democratic Republic of Ethiopia allows termination of pregnancy under some conditions [17]. Also Federal ministry of Health (FMoH) revised the technical and procedural guideline on June 2014 for safe abortion services for ascertaining quality of care and also allows first trimester pregnancy safe abortion care can be given at health center level as part of task sharing & task shifting [14].

Among respondents 71.9% knew national definition of abortion. This study showed the respondent had much better knowledge when compared with one study which was done in Tigray (63.3%) [18].It may be due to working place in capital town of the country.

Knowledge of the law is essential so that providers not only know what is expected of them but can also inform and educate women and community at large [14]. Majority of respondents (81.5%) were aware about the revised abortion law. However, only 85.2% of them knew that equipped health facilities with trained staffs that are authorized to perform the procedure. On other questions related to revised abortion law only 57.6% of respondents said, who claimed they were familiar about the revised abortion law, the woman who request termination of pregnancy are not required to submit evidence of rape or incest in order to obtain abortion service according to Penal code of FDRE though 31.5% of respondents said they would not give the service unless she submitted evidences. On other hand if continuation of pregnancy endangers the life woman or the child 69.4% participants said they will provide the SAC without her state of illness. The law does not require women to provide evidence for seeking safe abortion service following rape or incest, and shouldn’t be necessary ill if the pregnancy endangers her life or the child.

The provider, as mentioned in the penal code of FDRE, should get clear standard written consent information from all pregnant women who undergoing pregnancy termination after having an objective counseling [17]. The information should be clear, objective, and non-coercive and provided in a language understandable to the client. From this study, 84.2% of the participants had or would have access to a written consent from the woman before practising the safe abortion service which is lower when comparing a research done in Tigray (93%) [18].

Post –procedure care is essential as care during procedure to ensure maximum outcome in abortion care services. The post-abortion care (PAC) components are Community and service provider partnership, treatment of incomplete and complication of unsafe abortion, counseling, contraceptive and family planning service and integration of reproductive and other health services [14]. From this study participants (85.7%) knew the PAC, though 33.1% of respondents knew treatment of incomplete & complication of unsafe abortion and integration of reproductive & other health services as components of PAC which is less comparing from other studies (58.5%, 58) in Tigray and Addis Ababa respectively [18, 19].

As professionals, health care workers must learn to separate their personal beliefs and values from their professional practices and treat all women equally and with empathy, regardless of their reproductive behaviors and decisions [1, 8, 12].

The present study tried to obtain information on liberalization of abortion at any circumstances. Attitude favoring abortion to be legal was found to be 27.7% of which much lower than a study done on health providers in Addis Ababa health facilities (41.8%) [19]. Respondents’ reasons why they disagreed on liberalization of abortion were their religion doesn’t allow, it encourages pre−/extra-marital sex, encourages to have unwanted pregnancy and it is homicide on the fetus.

The national guideline under the subtitle of “provider’s skills and performance” clearly underlines the importance of providing basic knowledge and skills to health providers on regular basis in order to maximize their effectiveness to provide the service and manage abortion and its complications [14].

From this study only 20.5% took training on safe abortion; of them 81.9% applied their training on practice. This study showed almost similar results conducted previously in Addis Ababa (29.4%) in 2008 [19] and Tigray (20%) in 2011 have had the training [18]. This, unequivocally, suggests the need to introduce procedures of pregnancy termination during health service providers’ pre-service training. Among procedures majority practiced mifepristone with misoprostol and MVA. This finding congruent with guideline recommends health care providers should practice medication abortion for first trimester termination of pregnancy.

Repeated unwanted pregnancies and abortions are prevented by post abortion family planning (PAFP) counseling and service provision which has all elements of family planning, and can be offered at the abortion-care facility [14]. From our study half of providers (49.6%) offered post abortion family planning. Even all health professions expected to give post abortion family planning according to the guideline.

This study tried to assess factors which affect providers’ knowledge by taking knowledge score fitted to logistic regression. More than half (53.1%) of respondents had adequate knowledge related to abortion. The finding from the study showed midwives had better knowledge 2.9 times followed by health officers 2.7 times then nurses related to abortion. Midwives had better knowledge than other mid-level providers (MLPs) may be due to their daily activity is with women and pregnant mothers who help them to have better understanding related abortion. So, nurses need to have much pre−/on-service training and the curriculum also should focus on thus professions.

The other factor which has impact on MLPs knowledge related to abortion was their years of work experiences. The finding from this research showed providers working for 3–5 years had better knowledge 3 times followed by work experience of more than five years (2.8 times) than three years’ of experience. From this study as years of experiences increased their knowledge related to abortion decreases, so there should be a periodic update on abortion for professions.

This study also tried to fit logistic regression, by taking mean attitude score as the outcome variable, in order to disentangle the factors shaping the attitude of MLPs included in the study. From this study more than half (54.1%) of respondents had positive attitude towards safe abortion. The finding from the regression shows males were 1.6 times likely to have positive attitudes towards safe abortion than females. MLPs who had adequate knowledge related abortion were 2.2 likely to have a good attitude towards safe abortion than didn’t have adequate knowledge. On other studies, providers who had good knowledge about abortion were 6.9 times likely to have positive attitude towards safe abortion [20]. So, every effort should be tried by governmental and non-governmental institutions to increase MLPs knowledge related abortion to have a favorable response on safe abortion which is a crucial influence for a women to get the services.

From this study, knowledge related to abortion consistently influence MLPs attitude towards safe abortion and their practice of SAC. Effort should be done to maximize mid-level providers’ knowledge related to abortion by pre−/on-service trainings.

The findings of this research provide valuable information to guide efforts on the quality and access to abortion services in the country. However, this research only assessed MLPs’ KAP towards SAC. It would be better to include observing the availability of equipment and supplies and directly evaluating quality of SAC in the sample health centers. Also, participants were from health centers only; private institutions and government hospitals were not included and caution is needed in using the results of the study.

## Conclusion

More than half of respondents had adequate knowledge related to abortion. The majority claimed to know the current abortion law; however, many failed to understand the specific provisions of the Law.

Only half of participants offered post abortion family planning.

Being midwife and work experiences of 3–5 years were important in explaining providers’ knowledge related to abortion. Being male and having the knowledge on abortion significantly influenced providers’ attitude toward safe abortion. Knowledge related to abortion also influenced the practice of SAC.

Efforts to improve mid-level providers’ knowledge on abortion are necessary, for example, through pre−/on-service training.

Health facilities and providers should work according to the revised law of abortion and national technical & procedural guideline on safe abortion.

Further research including qualitative methods related to this topic at all health institutions among all professionals is recommended.

## Supplementary information

**Additional file 1.** Study questionnaire.

## Data Availability

The data will be available by requesting corresponding author.

## Abbreviations

D &C: Dilation and curettage
E &C: Evacuation and curettage
FDRE-MOH: Federal Democratic Republic of Ethiopia -Ministry of Health
IRB: Institutional Review Board
IUD: Intrauterine device
MLPs: Mid-level providers
MVA: Manual Vacuum Aspiration
OCP: Oral contraceptive pills
PAC: Post abortion care
SAC: Safe abortion care
