# Supplementary material for: Knowledge, attitude and practice (KAP) of health providers towards safe abortion provision in Addis Ababa health centers
Source: BMC Womens Health. 2019 Nov 14;19:138. doi: 10.1186/s12905-019-0835-x (PMC6854666; doi:10.1186/s12905-019-0835-x)
Supplement: Supplementary file 1 — Additional file 1. Study questionnaire. [file 12905_2019_835_MOESM1_ESM.docx]

**CONSENT FORM**

How are you, I am Endalkachew Mekonnen (MD). I would like to ask you few questions and your willingness in the study is essential. It is a self-administrative questionnaire.

This study is prepared to obtain relevant information about knowledge, attitude and practice of health providers towards safe abortion provision in Addis Ababa health centers. Your participation in the study is very important in reducing the maternal morbidity and mortality rate, which is caused by unsafe abortion and its complication.

Your name & address will not be written in this form and will never be used in connection with any information you tell us. All the information given by you will be kept strictly confidential and only used for this study. Your participation is voluntary and you are not obligated to answer any question which you do not wish to answer. If you fill discomfort to respond to any of the question, please fill free to drop it any time you wish to do so.

I have read all the process and the objective of the study and I have understood the same as written. I understood that the research imposes no risk would be provided to me and families.

Could I have your permission to continue?

1. Yes

2. No

The principal Investigator address: phone No. +251-913605674

[E-mail= endmekon@gmail.com](mailto:E-mail=%20endmekon@gmail.com), P.O.Box =9086

Date of Data Collection: __________ Name of Health Facility ________________

Sub-city ____________ Health Facility Code ______________

Result: Questionnaire completed _________________________

Questionnaire partially completed ________________

Participant refused ______________________________

Checked by Supervisor: Name _____________________________________

Supervisor’s Signature _______________________ Date ________________

**Questionnaires**

Section – one**: Demographic information**

| Serial No. | Questions | Response | Code | Skip |
| --- | --- | --- | --- | --- |
| 101 | Your age in completed years(enter number) | _____ years |  |  |
| 102 | Sex | 1. Female 2. Male | /____ / |  |
| 103 | Marital status | 1. Never married  2.Married  3.Divorced  4.Cohabiting  4.Separated  5.Widowed | / ____ / |  |
| 104 | Religion | 1.Orthodox  2.Muslim  3.Protestant  4.Catholic  5.Other (Specify) _________ | / _____ / |  |
| 105 | Profession | 1. Nurse (diploma) 2. B.Sc. nurse 3. Midwife (diploma) 4. Midwife(BSc) 5. Health Officer 6. Others (specify) ___________ | /_____ / |  |
| 106 | Years of professional experience | 1. Less than one year  2.one-three years  3. three -five years  4. five-ten years  5.More than 10 years | / _____ / |  |
| 107 | For how long have you been working in this health facility? | _________ year/s |  |  |

**Section two**: KNOWLEDGE OF HEALTH PROFFESTIONALS ON SAFE ABORTION CARE

| S no | Questions | Response | Code | Skip |
| --- | --- | --- | --- | --- |
| 2.1. | What is abortion? | 1. It is termination of pregnancy before fetal viability less than 20 weeks from LNMP  2.. It is termination of pregnancy before fetal viability less than 24 weeks from LNMP  3.It is termination of pregnancy before fetal viability less than 28 weeks from LNMP  4.I don’t know |  |  |
| 2.2. | Do you know safe abortion? | 1.Yes  2.No |  | If no skip No. 2.9 |
| 2.3 | What type of abortion procedure  do you know(more than one answer possible) | 1 .D&C  2. E&C  3.MVA  4. Using mifepristone & Misoprostol  5. I don’t know  6.other Specify ___________ |  |  |
| 2.4 | Are you trained for safe abortion care | 1.Yes  2.No |  | If no skip to no. 2.9 |
| 2.5 | If trained are you practicing it? | 1.Yes  2.No |  | If no skip to no. **2.8** |
| 2.6 | If you are practicing it, when did you perform safe termination of pregnancy? | 1. I’m currently practicing  2.within the last six months  3.b/n the last one and two years  4.before two years |  |  |
| 2.7 | Currently what type of procedures you are practicing?(more than one answer possible) | 1.Manual vacuum aspiration(MVA)  2.Medication abortion (mifepristone+ misoprostol)  3.Oxytocin induction  4.Dilatation and curettage(D&C )  5.Sharp curettage (E& C)  6.Other modalities(specify) _________ |  |  |
| 2.8 | If your answer is NO for No.2.5, why didn't you perform safe termination of pregnancy? (more than one answer possible) | 1. No women have sought this service  2. The facility doesn’t offer safe abortion services  3. Lack of equipment and Supply  4. The facility does not allow us to provide safe termination of pregnancy  5. Personal reason  6. Overload of work  7. I don’t know  8.Any other reasons(specify) _____________ |  |  |
| 2.9 | Are you familiar with the revised abortion law of Ethiopia? | 1. Yes 2. No |  | If No go to no. 2.16 |
| 2.10 | Where is the place for terminating pregnancy as permitted by the revised abortion law | 1. Equipped health facilities that are not authorized to perform the procedure with no trained staffs 2. Non-equipped health facilities that are not authorized to perform the procedure with no trained staffs 3. Equipped health facilities with trained staffs that are authorized to perform the procedure   4. I don’t know |  |  |
| 2.11 | What is required from a woman who requests termination of pregnancy due to rape or incest? | 1. The woman who request termination of pregnancy are required to submit evidence of rape or incest in order to obtain abortion service 2. The woman who request termination of pregnancy are not required to submit evidence of rape or incest in order to obtain abortion service   3. I don’t know |  |  |
| 2.12 | If the continuation of the pregnancy endangers the life of woman or the child in which state should be safe abortion permitted? | 1. The woman should necessarily be in a state of ill health at the time of requesting safe abortion services 2. The woman should not necessarily be in a state of ill health at the time of requesting safe abortion services   3. I don’t know  4. Others(specify) |  |  |
| 2.13 | The provider has to secure on informed consent for procedure using a standard consent form | 1. True 2. False |  |  |
| 2.14 | The health care institution and the health worker who provides the service has an ethical obligation not to disclose the information provided by the woman unless permitted by the woman or ordered by the court | 1. True 2. False |  |  |
| 2.15 | How many days do you think the timing is for a woman who is eligible for pregnancy termination to obtain the service? | 1. Within five working days 2. Within seven working days 3. Within three working days 4. I don’t know |  |  |
| 2.16 | Do you know the components of Post abortion care (PAC)? | 1. Yes 2. No |  | If NO skip to no. 2.18 |
| 2.17 | Which components do you know? (more than one choice possible) | 1. Community and service provider partnership 2. Counseling 3. Treatment of incomplete and complication of unsafe abortion 4. Contraceptive and FP service 5. Integration of Reproductive and other health Service 6. I don’t know |  |  |
| 2.18 | Who do you think can perform the procedure of MVA according to the revised technical and procedural guideline issued by Federal Ministry of Health(FMoH)?(more than choice is possible) | 1. Physicians/GPs 2. Health Officer 3. Midwives 4. nurses   5. I don’t know |  |  |
| 2.19 | Who do you think can perform medication abortion up to 9 weeks of pregnancy according to the revised technical and procedural guideline issued by FMoH?(more than choice is possible) | 1. Physicians/GPs  2. Health Officer  3. Midwives  4. Nurses  5. I don’t know |  |  |
| 2.20 | Who do you think can perform the procedure of Sharp Metallic curettage (SMC) for pregnancy termination according the technical and procedural guideline issued by FMoH? | 1. Junior nurse or health assistant 2. Medical doctor or gynecologist 3. Health officer 4. Nurse or midwife   88. I don’t know |  |  |
| 2.21 | Who do you think authorized to perform 2^nd^ trimester abortion procedures with adequate training (more than one choice is possible) | 1. specialists in Obstetrics-gynecology  2. GPs  3. Health officers  4. midwives  5. nurses  6. I don’t know |  |  |
| 2.22 | Referral arrangement for social support and care are an integral part of overall abortion care. | 1. True 2. False |  |  |
| 2.23 | Health centers expected to give 1^st^ trimester safe abortion services according to the revised technical and procedural guideline issued by FMoH. | 1. true 2. false |  |  |
| 2.24 | Whom do think can provide education of legal provision for abortion?(more than one choice possible) | 1. Physicians/GPs 2. Health Officer 3. Midwives 4. Clinical nurses 5. 5. I don’t know |  |  |
| 2.25 | Who should provide post abortion contraception?(more than one answer possible) | 1. Physicians/GPs   1. Health Officer 2. Midwives 3. Clinical nurses 4. I don’t know |  |  |
| 2.26 | Did you offer family planning methods after abortion? | 1. yes 2. no |  | If no skip to 2.28 |
| 2.27 | If yes, which method? | 1. Condom  2. Diaphragm or Cervical Cap  3. Oral Contraceptives  4. Injectables  5. Implant (Norplant)  6. IUD  7.natural methods  8.Other(specify)____________ |  |  |
| 2.28 | If no ,why | 1. I’m busy by other works  2. it isn’t my job  3. no family planning services in the health center  4. clients refused the services  5. I don’t know |  |  |

**Section three: ATTITUDES OF HEALTH PROFFESTIONALS ON SAFE ABORTION**

| S no | **Questions** | **Response** | code | skip |
| --- | --- | --- | --- | --- |
| 3.1 | Why do you think women seek abortion? | 1.Inadequate Knowledge  2.Economical constraint  3. Used as a Contraceptive  4.To avoid unwanted pregnancy  5. Health reasons  6. Partner pressure  7. Too many and too close pregnancies  8. To complete their education  9. Not being married  10. Other(specify)________________  11. I Don’t know |  |  |
| 3.2 | Do you feel comfortable working in a site where termination of pregnancy is being performed? | 1.Yes  2.No |  | If yes skip to 3.4 |
| 3.3 | If your answer is No, What is/are your reason/s? | 1. Outside of the scope of my practice  2. Against my religious practice  3. Against my Personal value  4. I didn’t have the opportunity to be  trained in abortion technique  5. I don’t know  6. Other(specify)_________ |  |  |
| 3.4 | Do you agree on the current legislation and institutional regulation of termination of pregnancy? | 1.Yes  2.No  3. I Don’t know |  |  |
| 3.5 | Elective abortion should be legal and  accessible under any circumstance | 1.Agree  2.Neutral  3.Disagree  4.Other Specify______________ |  |  |
| 3.6 | For whom do you think you will give safe abortion? (more than one answer possible) | 1. pregnancy following rape  2. pregnancy following incest  3. when continuation of the pregnancy endangers the health or life of the woman or the fetus  4. for women with physical or mental disabilities  5. if she is under 18 years or minor who are physically or psychologically unprepared to raise a child  6.in cases of fetal congenital anomaly incompatible for life |  |  |
| 3.7 | Legal abortion is used as a form of contraception. | 1.Agree  2 Disagree.  3. Neutral |  |  |
| 3.8 | Legal abortion should be permitted  under any circumstances | 1.Agree  2. Disagree.  3. Neutral |  |  |
| 3.9 | If you do disagree, what is your reason? | 1.My religion doesn’t allow  2.Culturally not accepted  3.It is homicide on the fetus  4.Encourages to have unwanted  Pregnancies  5.Encourages pre/extra- marital sex  6. I don’t know  7.others(specify)_________ |  |  |
| 3.10 | I am more comfortable with medical  abortion than surgical abortion for first trimester pregnancy | 1.Agree  2. Disagree  3. Neutral |  |  |
| 3.11 | Mid-level health providers should be able to provide medical abortion for first trimester pregnancy? | 1. Agree  2. Disagree  3. Neutral |  |  |
| 3.12 | Mid-level health providers should be able to provide surgical abortion for first trimester pregnancy | 1.Agree  2. Disagree  3. Neutral |  |  |

Additional comments if you have any:

Thank you, taking your time to fill the questioner.
